# SARS-CoV-2 Infections and Hospitalizations Among Persons Aged ≥16 Years, by Vaccination Status — Los Angeles County, California, May 1–July 25, 2021

**DOI:** 10.15585/mmwr.mm7034e5

**Published:** 2021-08-27

**Authors:** Jennifer B. Griffin, Meredith Haddix, Phoebe Danza, Rebecca Fisher, Tae Hee Koo, Elizabeth Traub, Prabhu Gounder, Claire Jarashow, Sharon Balter

**Affiliations:** ^1^Acute Communicable Disease Control Program, Los Angeles County Department of Public Health, California; ^2^Vaccine Preventable Disease Control Program, Los Angeles County Department of Public Health, California.

COVID-19 vaccines fully approved or currently authorized for use through Emergency Use Authorization from the Food and Drug Administration are critical tools for controlling the COVID-19 pandemic; however, even with highly effective vaccines, a proportion of fully vaccinated persons will become infected with SARS-CoV-2, the virus that causes COVID-19 (*1*). To characterize postvaccination infections, the Los Angeles County Department of Public Health (LACDPH) used COVID-19 surveillance and California Immunization Registry 2 (CAIR2) data to describe age-adjusted infection and hospitalization rates during May 1–July 25, 2021, by vaccination status. Whole genome sequencing (WGS)–based SARS-CoV-2 lineages and cycle threshold (Ct) values from qualitative reverse transcription–polymerase chain reaction (RT-PCR) for two SARS-CoV-2 gene targets, including the nucleocapsid (N) protein gene region and the open reading frame 1 ab (ORF1ab) polyprotein gene region,* were reported for a convenience sample of specimens. Among 43,127 reported SARS-CoV-2 infections in Los Angeles County residents aged ≥16 years, 10,895 (25.3%) were in fully vaccinated persons, 1,431 (3.3%) were in partially vaccinated persons, and 30,801 (71.4%) were in unvaccinated persons. Much lower percentages of fully vaccinated persons infected with SARS-CoV-2 were hospitalized (3.2%), were admitted to an intensive care unit (0.5%), and required mechanical ventilation (0.2%) compared with partially vaccinated persons (6.2%, 1.0%, and 0.3%, respectively) and unvaccinated persons (7.6%, 1.5%, and 0.5%, respectively) (p<0.001 for all comparisons). On July 25, the SARS-CoV-2 infection rate among unvaccinated persons was 4.9 times and the hospitalization rate was 29.2 times the rates among fully vaccinated persons. During May 1–July 25, the percentages of B.1.617.2 (Delta) variant infections estimated from 6,752 samples with lineage data increased among fully vaccinated persons (from 8.6% to 91.2%), partially vaccinated persons (from 0% to 88.1%), and unvaccinated persons (from 8.2% to 87.1%). In May, there were differences in median Ct values by vaccination status; however, by July, no differences were detected among specimens from fully vaccinated, partially vaccinated, and unvaccinated persons by gene targets. These infection and hospitalization rate data indicate that authorized vaccines were protective against SARS-CoV-2 infection and severe COVID-19 during a period when transmission of the Delta variant was increasing. Efforts to increase COVID-19 vaccination, in coordination with other prevention strategies, are critical to preventing COVID-19–related hospitalizations and deaths.

LACDPH analyzed data for laboratory-confirmed cases of SARS-CoV-2 reported from testing laboratories to LACDPH during May 1–July 25, 2021, which included a total of 9,651,332 Los Angeles County residents (excluding Pasadena and Long Beach residents).^†^ A laboratory-confirmed SARS-CoV-2 infection was defined as a first detection^§^ of SARS-CoV-2 RNA or antigen in a respiratory specimen. Vaccination status was ascertained by matching SARS-CoV-2 case surveillance and CAIR2 data on person-level identifiers using an algorithm with both deterministic and probabilistic passes. Persons were considered fully vaccinated ≥14 days after receipt of the second dose in a 2-dose series (Pfizer-BioNTech or Moderna COVID-19 vaccines) or after 1 dose of the single-dose Janssen (Johnson & Johnson) COVID-19 vaccine^¶^; partially vaccinated ≥14 days after receipt of the first dose and <14 days after the second dose in a 2-dose series; and unvaccinated <14 days after receipt of the first dose of a 2-dose series or 1 dose of the single-dose vaccine or if no CAIR2 vaccination data were available. COVID-19–associated hospitalizations were defined as hospital admissions occurring ≤14 days after a first SARS-CoV-2 infection. COVID-19–associated deaths were defined as deaths occurring ≤60 days after the date of a first laboratory-confirmed SARS-CoV-2 infection or deaths with COVID-19 listed as a cause of or contributing condition to death.

Differences in the percentages of infections by vaccination status were calculated using chi-square tests for categorical variables and Kruskal-Wallis tests for medians; p-values <0.05 were considered statistically significant. Age-adjusted rolling 7-day SARS-CoV-2 infection and hospitalization rates were estimated by vaccination status.** Using convenience samples, WGS lineage data from all available sequencing results (6,752)^††^ and Ct values from diagnostic qualitative RT-PCR assays targeting two genes (SARS-CoV-2 nucleocapsid [SC2N; 5,179], ORF1ab [1,041], and N [1,062]) from two laboratories were reported over time by vaccination status. Analyses were conducted using SAS (version 9.4; SAS Institute). This activity was determined by LACDPH’s Institutional Review Board (IRB) to be a surveillance activity necessary for public health work and therefore did not require IRB review.

The percentage of fully vaccinated Los Angeles County residents increased from 27% on May 1, 2021, to 51% on July 25, 2021. During the same period, 43,127 cases of SARS-CoV-2 infection among residents aged ≥16 years were reported to LACDPH, including 10,895 (25.3%) in fully vaccinated persons, 1,431 (3.3%) in partially vaccinated persons, and 30,801 (71.4%) in unvaccinated persons (Table). The largest percentages of cases across all groups were among adults aged 30–49 years and 18–29 years, females, and Hispanic persons. Among fully vaccinated persons on July 25, 55.2% had received the Pfizer-BioNTech vaccine, 28.0% had received the Moderna vaccine, and 16.8% had received the Janssen vaccine. Lower percentages of fully vaccinated persons were hospitalized (3.2%), were admitted to an intensive care unit (0.5%), and required mechanical ventilation (0.2%) compared with partially vaccinated persons (6.2%, 1.0%, and 0.3%, respectively) and unvaccinated persons (7.6%, 1.5%, and 0.5%, respectively) (p<0.001). Among hospitalized persons and persons admitted to an intensive care unit, the median age was higher among vaccinated persons (median = 64 years, interquartile range [IQR] = 53.0–76.0 years; median = 64 years, IQR = 54.0–76.0 years, respectively) and partially vaccinated persons (median = 59, IQR = 46.0–72.0; median = 65, IQR = 57.0–80.0, respectively) than among unvaccinated persons (median = 49, IQR = 35.0–62.0; median = 56, IQR = 41.0–66.0, respectively) (p<0.001). A lower percentage of fully vaccinated (1.2%) and partially vaccinated (2.0%) persons were admitted to a hospital after their SARS-CoV-2 positive test result date compared with unvaccinated persons (4.2%). A lower percentage of deaths (0.2%, 24) occurred among fully vaccinated persons than among partially vaccinated (0.5%, seven) and unvaccinated (0.6%, 176) persons (p<0.001). Death investigations determined that six of the 24 fully vaccinated persons who died had immunocompromising conditions, including HIV infection, cancer (i.e., prostate, pancreatic, lung, or leukemia), and liver transplantation, and that the median age was higher among vaccinated (median = 78 years, IQR = 63.5–87.5 years) and partially vaccinated (median = 74, IQR = 58.0–80.0) persons than among unvaccinated persons (median = 63, IQR = 51.5–79.5) (p = 0.01).

**TABLE:** Number of SARS-CoV-2 cases among persons aged ≥16 years, by selected characteristics and vaccination status* — Los Angeles County, California,^†^ May 1–July 25, 2021

Characteristic	Vaccination status, no. (%)				p-value
	Total	Fully vaccinated	Partially vaccinated	Unvaccinated	
**Total no. of cases**	**43,127**	**10,895**	**1,431**	**30,801**	—
**Vaccine manufacturer**					
Janssen (Johnson & Johnson)	**—**	1,830 (16.8)	—	—	—
Moderna	**—**	3,047 (28.0)	—	—	—
Pfizer-BioNTech	**—**	6,018 (55.2)	—	—	—
**Median interval between final vaccine dose and infection, no. of days (IQR)**	**—**	98 (74–120)	—	—	—
**Median age, yrs (IQR)**	**34 (26–46)**	37 (28–52)	35 (27–51)	32 (26–44)	<0.001
**Age group, yrs**					
16–17	**1,120 (2.6)**	107 (1.0)	34 (2.4)	979 (3.2)	<0.001
18–29	**14,758 (34.2)**	3,017 (27.7)	432 (30.2)	11,309 (36.7)	
30–49	**18,106 (42.0)**	4,649 (42.7)	582 (40.7)	12,875 (41.8)	
50–64	**6,418 (14.9)**	2,025 (18.6)	255 (17.8)	4,138 (13.4)	
65–79	**2,101 (4.9)**	857 (7.9)	95 (6.6)	1,149 (3.7)	
≥80	**624 (1.4)**	240 (2.2)	33 (2.3)	351 (1.1)	
**Sex**					
Female	**21,743 (50.4)**	5,514 (50.6)	757 (52.9)	15,472 (50.2)	<0.001
Male	**20,425 (47.4)**	5,249 (48.2)	659 (46.1)	14,517 (47.1)	
Other or unknown	**959 (2.2)**	132 (1.2)	15 (1.0)	812 (2.6)	
**Race/Ethnicity**					
American Indian or Alaska Native	**70 (0.2)**	17 (0.2)	2 (0.1)	51 (0.2)	<0.001
Asian	**1,970 (4.6)**	905 (8.3)	104 (7.3)	961 (3.1)	
Black or African American	**5,574 (12.9)**	681 (6.3)	138 (9.6)	4,755 (15.4)	
Hispanic or Latino	**14,144 (32.8)**	3,450 (31.7)	511 (35.7)	10,183 (33.1)	
Multiple race	**823 (1.9)**	272 (2.5)	32 (2.2)	519 (1.7)	
Native Hawaiian or Other Pacific Islander	**210 (0.5)**	41 (0.4)	8 (0.6)	161 (0.5)	
Other	**3,998 (9.3)**	778 (7.1)	112 (7.8)	3,108 (10.1)	
White	**9,338 (21.7)**	3,397 (31.2)	321 (22.4)	5,620 (18.2)	
Missing	**7,000 (16.2)**	1,354 (12.4)	203 (14.2)	5,443 (17.7)	
**Hospitalized**	**2,794 (6.5)**	350 (3.2)	89 (6.2)	2,355 (7.6)	<0.001
**Admitted to ICU**	**536 (1.2)**	55 (0.5)	15 (1.0)	466 (1.5)	<0.001
**Required mechanical ventilation**	**189 (0.4)**	19 (0.2)	5 (0.3)	165 (0.5)	<0.001
**Admitted to hospital after positive SARS-CoV-2 test date**	**1,454 (3.4)**	136 (1.2)	29 (2.0)	1,289 (4.2)	<0.001
**Died**	**207 (0.5)**	24 (0.2)	7 (0.5)	176 (0.6)	<0.001

**Abbreviations:** ICU = intensive care unit; IQR = interquartile range.

* Persons were considered fully vaccinated ≥14 days after receipt of the second dose in a 2-dose series (Pfizer-BioNTech or Moderna COVID-19 vaccines) or after 1 dose of the single-dose Janssen (Johnson & Johnson) COVID-19 vaccine; partially vaccinated ≥14 days after receipt of the first dose and <14 days after the second dose in a 2-dose series; and unvaccinated <14 days receipt of the first dose of a 2-dose series or 1 dose of the single-dose vaccine or if no vaccination registry data were available.

^†^ Among residents of Los Angeles County; excludes Pasadena and Long Beach.

Among all Los Angeles County residents, the age-adjusted 7-day incidence and hospitalization rates increased exponentially among unvaccinated, fully vaccinated, and partially vaccinated persons, with the highest rates among unvaccinated persons in late June (Figure 1). On May 1, in unvaccinated persons, the age-adjusted incidence (35.2 per 100,000 population) was 8.4 times and the age-adjusted hospitalization rate (4.6 per 100,000 population) was 10.0 times the rates in fully vaccinated persons (4.2 and 0.46, respectively). Partially vaccinated persons had a similar incidence (4.1) and hospitalization rate (0.27) as fully vaccinated persons. On July 25, the age-adjusted incidence in unvaccinated persons (315.1) was 4.9 times that in fully vaccinated persons (63.8); the rate among partially vaccinated persons was 46.8. The age-adjusted hospitalization rate in unvaccinated persons (29.4) was 29.2 times the rate in fully vaccinated persons (1.0); the hospitalization rate was similar in partially vaccinated persons (0.90) (Supplementary Table; https://stacks.cdc.gov/view/cdc/109087).

**FIGURE 1:**
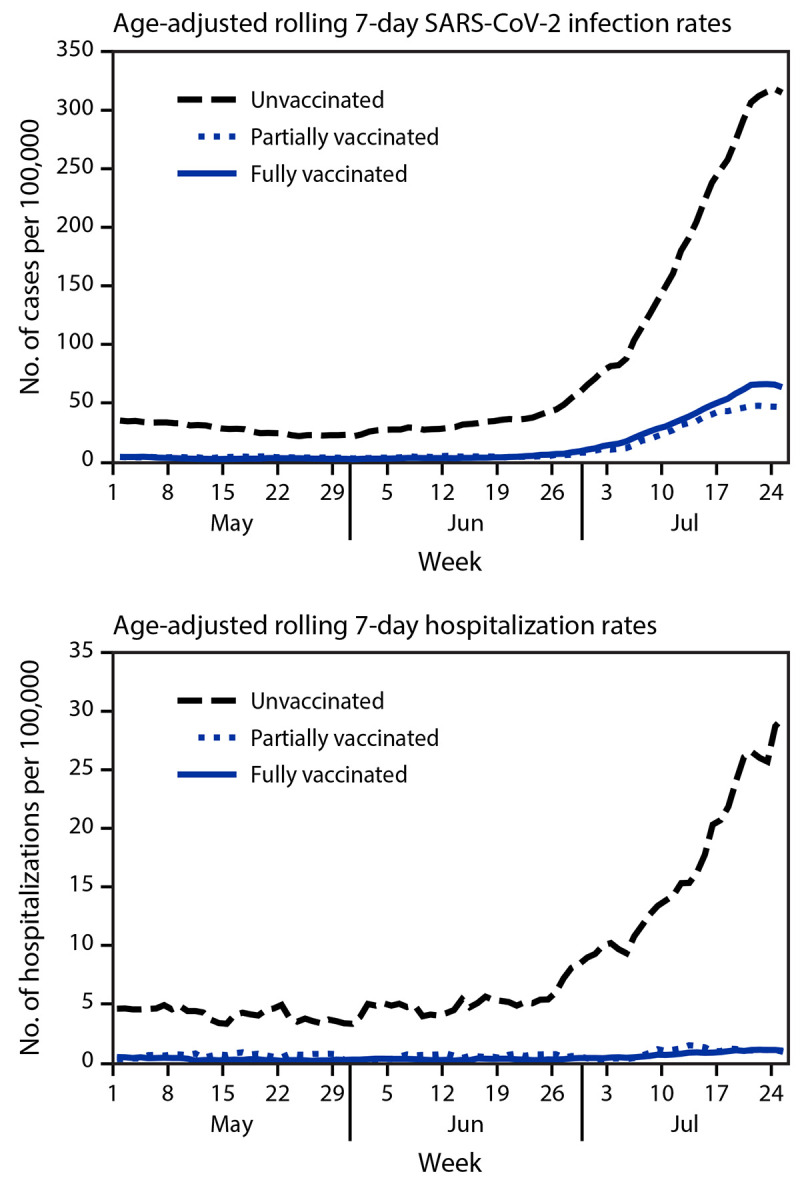
Age-adjusted rolling 7-day SARS-CoV-2 infection and hospitalization rates,* by vaccination status^†^ — Los Angeles County, California, May 1–July 25, 2021 * Rolling 7-day incidence was calculated by summing the total number of persons or hospitalizations during a 7-day period and dividing by the total population at the end of the 7-day period. ^†^ Persons were considered fully vaccinated ≥14 days after receipt of the second dose in a 2-dose series (Pfizer-BioNTech or Moderna COVID-19 vaccines) or after 1 dose of the single-dose Janssen (Johnson & Johnson) COVID-19 vaccine; partially vaccinated ≥14 days after receipt of the first dose and <14 days after the second dose in a 2-dose series; and unvaccinated <14 days receipt of the first dose of a 2-dose series or 1 dose of the single-dose vaccine or if no vaccination registry data were available.

During May 1–July 25, the percentages of residents aged ≥16 years with SARS-CoV-2 Delta variant infections increased from 8.6% to 91.2% in fully vaccinated persons (1,667), from 0% to 88.1% in partially vaccinated persons (198), and from 8.2% to 87.1% in unvaccinated persons (4,887) (Figure 2). In May, median Ct values were lower in specimens from unvaccinated persons than in those from partially vaccinated and fully vaccinated persons for the ORF1ab gene target (22.8, 36.6, and 27.7, respectively) and N gene target (24.0, 36.0, and 30.6, respectively); however, in July, no differences were found by vaccination status among the gene targets (SC2N = 19.3, 20.2, and 19.4; ORF1ab = 18.8, 17.8, and 19.0; N = 19.3, 18.6, and 19.5, respectively) (Figure 2).

**FIGURE 2:**
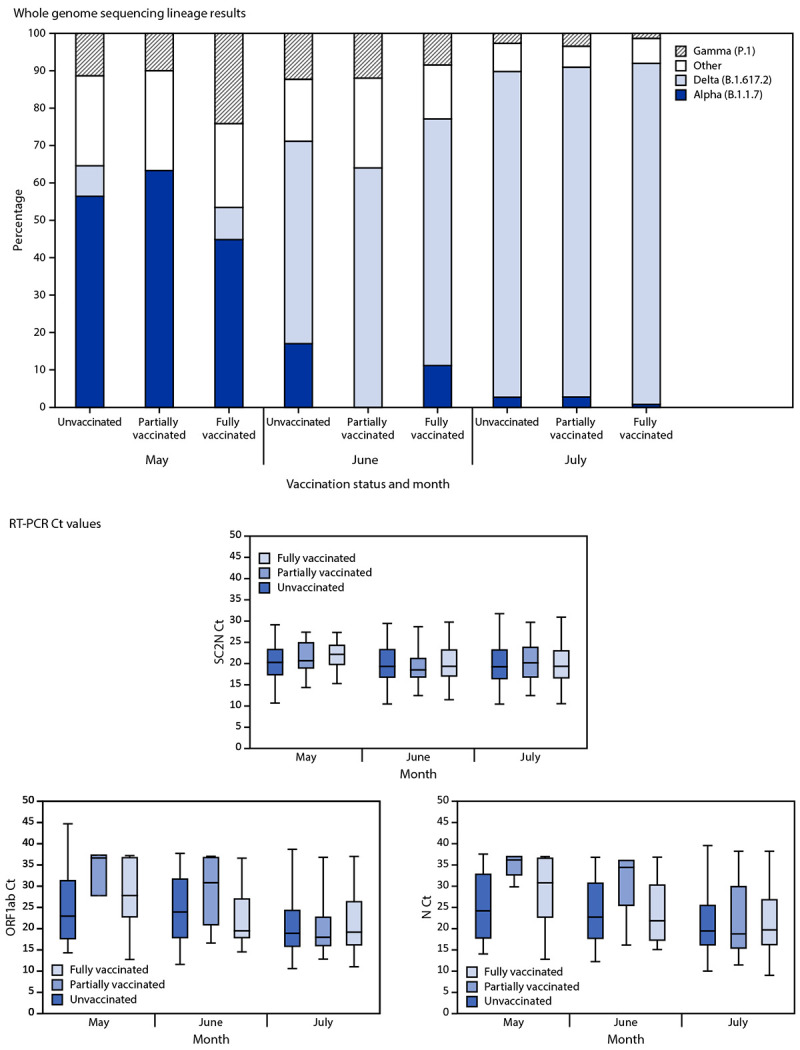
SARS-CoV-2 whole genome sequencing lineage results* and reverse transcription–polymerase chain reaction cycle threshold values^†^ for two gene targets,^§^ by vaccination status^¶^ and month — Los Angeles County, California, May 1–July 25, 2021 **Abbreviations:** Ct = cycle threshold; N = nucleocapsid; ORF1ab = open reading frame 1 ab; RT-PCR = reverse transcription–polymerase chain reaction; SC2N = SARS-CoV-2 nucleocapsid. * SARS-CoV-2 infections among Los Angeles County residents aged ≥16 years with whole genome sequencing lineage results (n = 6,752) for fully vaccinated (n = 1,667), partially vaccinated (n = 198), and unvaccinated (n = 4,887) persons. ^†^ Whiskers represent minimum and maximum observations; top of box represents the third quartile, bottom represents the first quartile, and box height represents the interquartile range. The midline is the median. ^§^ Ct values are correlated with the amount of viral nucleic acid present. Gene targets for RT-PCR testing included the N protein gene region and the ORF1ab polyprotein gene region. The N gene targets were analyzed separately for two laboratories because Ct values are not directly comparable across different testing laboratories; these N gene targets were designated SC2N and N to differentiate between the two participating laboratory partners. Gene targets were selected based on testing platforms used by Los Angeles County Department of Public Health laboratory partners. Analysis of SC2N Ct values is restricted to a Fulgent test result with a Ct value on the same day as person’s first positive RT-PCR test result; SC2N gene target values (n = 5,179) are stratified for fully vaccinated (n = 1,248), partially vaccinated (n = 151), and unvaccinated (n = 3,780) persons. Analysis of ORF1ab and N Ct values is restricted to a Valencia Branch Laboratory test result with a Ct value on the same day as person’s first positive RT-PCR test result. ORF1ab (n = 1,041) and N (n = 1,062) gene target values are stratified for fully vaccinated (n = 289 and n = 297, respectively), partially vaccinated (n = 36 and n = 41, respectively), and unvaccinated (n = 716 and n = 724, respectively) persons. ^¶^ Persons were considered fully vaccinated ≥14 days after receipt of the second dose in a 2-dose series (Pfizer-BioNTech or Moderna COVID-19 vaccines) or after 1 dose of the single-dose Janssen (Johnson & Johnson) COVID-19 vaccine; partially vaccinated ≥14 days after receipt of the first dose and <14 days after the second dose in a 2-dose series; and unvaccinated <14 days receipt of the first dose of a 2-dose series or 1 dose of the single-dose vaccine or if no vaccination registry data were available.

## Discussion

The results of this population-based analysis using linked SARS-CoV-2 infection surveillance and vaccination registry data indicate that fully vaccinated persons aged ≥16 years with SARS-CoV-2 infection were less likely than unvaccinated persons to be hospitalized, to be admitted to an intensive care unit, to require mechanical ventilation, or to die from SARS-CoV-2 infection during a period when the Delta variant became predominant. Although age-adjusted hospitalization rates in partially vaccinated persons were similar to those in fully vaccinated persons, age-adjusted incidences were slightly lower in partially vaccinated persons than in fully vaccinated persons. These data indicate that authorized vaccines protect against SARS-CoV-2 infection and severe COVID-19, even with increased community transmission of the newly predominant Delta variant (*2*).

The SARS-CoV-2 Delta variant is highly transmissible (*3*) and became the predominant variant in Los Angeles County during May–July 2021. During this period, SARS-CoV-2 cases and hospitalizations increased substantially, most notably among unvaccinated persons. In May, specimens from fully vaccinated and partially vaccinated persons had higher Ct values for two gene targets compared with unvaccinated persons; however, by July, median Ct values had decreased and were similar in all gene regions in specimens from fully vaccinated, partially vaccinated, and unvaccinated persons. These findings are similar to those from a recent study showing no difference in Ct values in specimens from vaccinated and unvaccinated persons during a large outbreak (*4*). Ct values are correlated with the amount of viral nucleic acid present; however, Ct values are an imperfect proxy for viral nucleic acid load, are not standardized across testing platforms, vary by specimen type and time from specimen collection, and should be limited to assessing differences at the population level, not the person level.^§§^

The findings in this report are subject to at least six limitations. First, vaccination data for persons who lived in Los Angeles County at the time of their laboratory-confirmed infection but who were vaccinated outside of California were unavailable, leading to misclassification of their vaccination status; if vaccinated persons without accessible records were considered to be unvaccinated, the incidence in unvaccinated persons could be underestimated. Second, case ascertainment is based on passive surveillance, with known underreporting that might differ by vaccination status. Similarly, screening and testing behaviors might differ among groups. Third, COVID-19–associated hospitalizations were determined based on hospital admission and SARS-CoV-2 test dates alone, leading to the inclusion of incidental hospitalizations that were not associated with COVID-19. Fourth, COVID-19–associated deaths included deaths occurring ≤60 days after a first SARS-CoV-2 positive test date; therefore, some COVID-19–associated deaths might have been from other causes (excluding trauma). In addition, certain COVID-19–associated deaths might have been a result of long-term sequelae after 60 days and were not included. Fifth, lineage and Ct values were available only for a convenience sample of SARS-CoV-2 cases. Finally, all the assays used to generate Ct values for comparison were qualitative, and none is approved for use in quantitating the amount of viral nucleic acid present.

The findings in this report are similar to those from recent studies indicating that COVID-19 vaccination protects against severe COVID-19 in areas with increasing prevalence of the SARS-CoV-2 Delta variant (*5*,*6*). Efforts to increase COVID-19 vaccination coverage, in coordination with other prevention strategies, are critical to preventing COVID-19–related hospitalizations and deaths. Ongoing surveillance to characterize postvaccination infections, hospitalizations, and deaths will be important to monitor vaccine effectiveness, particularly as new variants emerge.

**Summary**

**What is already known about this topic?**

Although COVID-19 vaccines are highly effective, some fully vaccinated persons will be infected with SARS-CoV-2.

**What is added by this report?**

During May 1–July 25, 2021, among 43,127 SARS-CoV-2 infections in residents of Los Angeles County, California, 10,895 (25.3%) were in fully vaccinated persons, 1,431 (3.3%) were in partially vaccinated persons, and 30,801 (71.4%) were in unvaccinated persons. On July 25, infection and hospitalization rates among unvaccinated persons were 4.9 and 29.2 times, respectively, those in fully vaccinated persons. In July, when the Delta variant was predominant, cycle threshold values were similar for unvaccinated, partially vaccinated, and vaccinated persons.

**What are the implications for public health practice?**

Efforts to enhance COVID-19 vaccination coverage, in coordination with other prevention strategies, are critical to preventing COVID-19–related hospitalizations and deaths.

## Footnotes

* Gene targets for RT-PCR testing included the N protein gene region and the ORF1ab polyprotein gene region. The N gene targets were analyzed separately for two laboratories because Ct values are not directly comparable across different testing laboratories; these N gene targets were designated SARS-CoV-2 nucleocapsid (SC2N) and N to differentiate between the two participating laboratory partners. Gene targets were selected based on testing platforms used by LACDPH laboratory partners.

† The population of Los Angeles County residents is based on 2018 population estimates prepared for Los Angeles County Internal Services Department. These population estimates exclude the populations of Pasadena and Long Beach because they have independent public health departments.

§ Cases were limited to first laboratory-confirmed infections and excluded reinfections.

¶
https://www.cdc.gov/coronavirus/2019-ncov/vaccines/fully-vaccinated-guidance.html

** Adjusted rates were calculated using 2018 population estimates and were standardized using the year 2000 U.S. standard population (https://www.cdc.gov/cancer/uscs/technical_notes/stat_methods/rates.htm). Rolling 7-day incidence was calculated by summing the total number of persons or hospitalizations during a 7-day period and dividing by the total population at the end of the 7-day period.

†† WGS lineage data were from all sequencing results reported to LACDPH or sequenced after specimens were referred to LACDPH laboratories.

§§ Additional information on Ct values and their limitations is available: https://www.idsociety.org/globalassets/idsa/public-health/covid-19/idsa-amp-statement.pdf and https://www.cdc.gov/coronavirus/2019-ncov/lab/faqs.html.
